# How previous experience shapes perception in different sensory modalities

**DOI:** 10.3389/fnhum.2015.00594

**Published:** 2015-10-31

**Authors:** Joel S. Snyder, Caspar M. Schwiedrzik, A. Davi Vitela, Lucia Melloni

**Affiliations:** ^1^Department of Psychology, University of NevadaLas Vegas, Las Vegas, NV, USA; ^2^Laboratory of Neural Systems, The Rockefeller UniversityNew York, NY, USA; ^3^Department of Neurophysiology, Max Planck Institute for Brain ResearchFrankfurt, Germany; ^4^Department of Neurological Surgery, Columbia UniversityNew York, NY, USA; ^5^Comprehensive Epilepsy Center, Department of Neurology, NYU Langone Medical Center, NYU School of Medicine, New York UniversityNew York, NY, USA

**Keywords:** context effects, adaptation, perceptual memory, Bayesian modeling, vision, audition

## Abstract

What has transpired immediately before has a strong influence on how sensory stimuli are processed and perceived. In particular, temporal context can have contrastive effects, repelling perception away from the interpretation of the context stimulus, and attractive effects (TCEs), whereby perception repeats upon successive presentations of the same stimulus. For decades, scientists have documented contrastive and attractive temporal context effects mostly with simple visual stimuli. But both types of effects also occur in other modalities, e.g., audition and touch, and for stimuli of varying complexity, raising the possibility that context effects reflect general computational principles of sensory systems. Neuroimaging shows that contrastive and attractive context effects arise from neural processes in different areas of the cerebral cortex, suggesting two separate operations with distinct functional roles. Bayesian models can provide a functional account of both context effects, whereby prior experience adjusts sensory systems to optimize perception of future stimuli.

## Introduction

The information our senses receive is often ambiguous, incomplete, and discontinuous. Nevertheless, we perceive our environment as a unified whole. According to a theory originally proposed by Helmholtz (1867), our brains achieve this using prior information. For example, when talking to someone, we decipher each word by taking into account not only the sounds and movements coming from the speaker’s mouth but also the meaning of preceding words, the topic of the conversation, as well as our lifelong knowledge of language. One particularly important type of experience that can aid perception is what has just occurred (i.e., *temporal context*). Temporal context effects (TCEs) are evident in various experimental phenomena, such as visual aftereffects. Yet, TCEs also occur in other sensory modalities, such as audition and touch. Given the pervasive influence that previous experience has on perception, it is essential to understand what factors determine how this perceptual adjustment occurs. Here, we explain TCEs using Bayesian theory, with an emphasis on how it explains TCEs across senses and levels of processing.

## TCEs in Perception

### Opposing Context Effects on Perception

**Figure 1:**
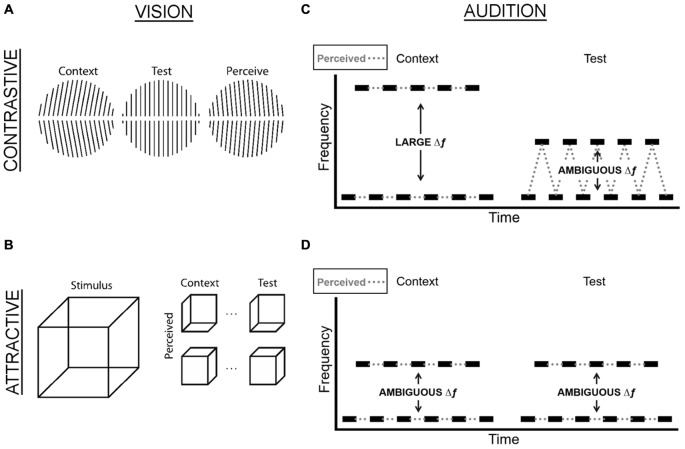
**Examples of contrastive and attractive TCEs in vision and hearing. (A)** In the famous tilt aftereffect, prolonged viewing of tilted lines (context) causes subsequently presented, straight lines (test) to be perceived as tilted in the opposite direction (perceive), a contrastive TCE. **(B)** When two ambiguous Necker cubes are presented in temporal succession, the perceived orientation of the first Necker cube (context) often determines the perceived orientation of the second Necker cube (test), although all interpretations are equally likely when the same Necker cube is presented in isolation. For example, if the first cube is perceived as facing up (down), this will provide the context for the second cube, which will be perceived as facing up (down), too. This stabilization of perception is an attractive TCE. **(C)** In the auditory stream segregation paradigm, the test tones have a constant ambiguous Δf throughout the duration of the experiment. When the Δf of the context is larger than the test, this typically leads to a contrastive effect, whereby listeners perceive the context as two separate streams and the test as one stream. **(D)** When the Δf of the context is the same ambiguous Δf as the test, this typically leads to listeners reporting that they hear the same percept as they had for the context (e.g., if they heard two streams for the context, they are more likely to hear two streams for the test).

Two TCEs have been investigated most extensively: the first typically occurs when a non-ambiguous, salient context stimulus (e.g., leftward tilted lines) precedes a test stimulus (e.g., vertically oriented lines), which results in perception being repelled away from the interpretation of the context stimulus such that participants perceive the test stimulus lines as tilted rightward (Figure 1A). Similarly, in the waterfall illusion, also known as the motion aftereffect, a rock on the side of the stream is usually perceived as moving upward after staring at the downward motion of the waterfall (Addams, 1834). This *contrastive* effect is known as *adaptation*, *negative aftereffect*, or *habituation*. There is considerable evidence that it results from neural adaptation, which in turn alters the balance of population activity, thus favoring perception of features that are not adapted (Grunewald and Lankheet, 1996; Huk et al., 2001).

The second type of effect is typically (Brascamp et al., 2007), but not always (Fischer and Whitney, 2014) observed when context and test stimuli are weak or ambiguous. For example, if two Necker cubes are presented sequentially, the percept of the first cube (e.g., face down) will typically dominate how the second cube is perceived (i.e., face down again), despite the fact that either perceptual interpretation of the cube is equally likely in isolation (Figure 1B). This *attractive* effect is known as *facilitation*, *perceptual* *memory*, *hysteresis*, or *stabilization* (Pearson and Brascamp, 2008). Although the term “priming” is also sometimes used, this can also refer to facilitation of reaction times (Pickering and Ferreira, 2008). Importantly, unlike the first (contrastive) effect, which is the result of *stimulus features* of the context, attractive effects depend more strongly on the *perceptual interpretation* of the context stimulus (Hock et al., 1996; De Lucia et al., 2010; Schwiedrzik et al., 2014; but see Kanai and Verstraten, 2005).

Why do these two context effects occur and why do they have opposite effects on perception? One possibility is that *attractive* and *contrastive* context effects serve different functions. Although there are many theories that explain why contrastive effects are so pervasive in perception, they mostly agree that contrastive effects sensitize the brain to take in new information, facilitating the detection of changes (Barlow, 1990; Clifford et al., 2000). On the other hand, attractive effects may stabilize perception in the face of noisy, discontinuous, and constantly changing environments brought about by eye and head movements, stimulus occlusion, or spontaneous neural fluctuations (Kleinschmidt et al., 2002). The coexistence of attractive and contrastive effects (Snyder et al., 2009; Denison et al., 2011; Schwiedrzik et al., 2014) may then endow the brain with the flexibility to deal with constantly changing internal and external demands, accomplishing balance between constancy and variation.

### General-Purpose Mechanisms Across Senses

TCEs also occur in senses other than vision (Riskey et al., 1979; Hulshoff Pol et al., 1998; Carter et al., 2008; Thiel et al., 2014). In audition, frequency glides cause similar aftereffects as those observed for visual motion, aftereffects, whereby a test stimulus—an illusory frequency modulation in a constant-frequency stimulus—is perceived as having the opposite direction as a context stimulus with periodic frequency modulation (Shu et al., 1993). As the motion aftereffect transfers between eyes (Anstis et al., 1998), this effect transfers between ears, suggesting that both effects arise from central motion processing. Attractive effects have been observed with ambiguous tritone stimuli (i.e., two tones that have no pitch height cues and form a six-semitone interval, Deutsch, 1997), by presenting tone pairs with increasing sized intervals for each successive pair starting with an ascending melodic interval (i.e., D#-E, D#-F, D#-F#, D#-G, … D#-D), or a descending melodic interval (i.e., D#-D, D#-C#, D#-C, D#-B, … D#-E; Giangrande et al., 2003). These ordered conditions biased the normally ambiguous tritone stimulus to be perceived as moving in the same direction as the initial small intervals.

Auditory scene analysis (Bregman, 1990; Snyder and Alain, 2007; Snyder et al., 2012), the perceptual segregation and integration of sound elements to form auditory objects, also shows contrastive and attractive context effects in experimental paradigms such as the stream segregation task. Listeners are presented with sequences of two tones of different frequencies in an alternating pattern, and the likelihood to perceive one or two streams depends on the frequency separation between the tones (Δf): the larger the separation, the *more* likely one hears two segregated streams. However, when a preceding context sequence has a larger Δf than the test, listeners are *less* likely to perceive the test sequence as two segregated streams—a *contrastive* effect (Figure 1C; Snyder et al., 2008, 2009; Snyder and Weintraub, 2011). An *attractive* effect of prior perception occurs during stream segregation tasks when context and test sequences have the same Δf (Figure 1D). The fact that the prior Δf effect generalizes to different frequency ranges (Snyder et al., 2009) but less so to different rhythmic patterns (Snyder and Weintraub, 2011) points to the recruitment of complex representations that are not arranged according to frequency. The contrastive effect declines over several seconds (Snyder et al., 2008; Snyder and Weintraub, 2013) and shows a persistent component (Snyder and Weintraub, 2013), suggesting the involvement of long auditory memory stores (Cowan, 1984; Lu et al., 1992). Importantly, the temporal dynamics of TCEs in audition can be strikingly similar to those in vision: for example, contrastive TCEs in visual apparent motion scale with log alternation rate (Anstis et al., 1985), as do contrastive effects in auditory stream segregation (Anstis and Saida, 1985). The fact that TCEs in vision and audition are not only phenomenologically similar but also both centrally based and share temporal properties suggests that the existence of attractive and contrastive context effects across domains reflects the likely operation of general-purpose mechanisms, achieving balance between perceptual constancy and variation. The shared properties of attractive and contrastive TCEs may originate from common computations by individual neurons or neural networks that similarly shape the processing of stimuli across domains.

### Context Effects with Complex Stimuli

Context effects are not limited to simple stimuli, such as tones or oriented lines, but are also prevalent for complex stimuli and can even occur across senses. In audition, a classic study demonstrated that listeners’ perception of a target word changed, for example, from a “bit” to a “bet”, depending on the acoustic characteristics of the preceding phrase (Ladefoged and Broadbent, 1957; also see Laing et al., 2012). Similarly, listeners perceive an ambiguous target word as either “bet” or “but” depending on the frequency content of the preceding context phrase (Huang and Holt, 2012). After exposure to a low-frequency version of the phrase, listeners more often hear “bet”, which typically contains more high-frequency energy; following a high-frequency version of the phrase, they more often hear “but”, which contains more low-frequency energy. Critically, the same patterns of contrastive effects are observed when the context consists of sequences of tones of similar frequency content to that of the phrase, suggesting a general computation that is not speech-specific. Speech context effects have been found to occur when context and target are presented dichotically (Holt and Lotto, 2002), suggesting involvement of cortical mechanisms. Contrastive effects have also been described in studies that manipulate speaking rate (Heffner et al., 2013) or style (casual vs. clear, Vitela et al., 2013) and with audiovisual stimuli: for example, presenting a face unambiguously articulating the syllable /aba/ or /ada/ decreases the likelihood to perceive the following unambiguous syllable as /aba/ or /ada/, respectively (Bertelson et al., 2003).

Attractive effects in speech perception have been reported for word identification (Tuller et al., 1994; Case et al., 1995) and in determining the meaning of a sentence through prosodic (or pitch) cues (Raczaszek et al., 1999). Again, attractive effects on speech perception are also observed across sensory modalities: brief exposure to a face articulating the syllable /aba/ or /ada/ increases the likelihood to perceive the identity of ambiguous auditory targets in the direction of the visual component (Bertelson et al., 2003; Vroomen et al., 2007). Attractive effects of similar magnitude and with a similar time course have also been reported for the perception of ambiguous phonemes when participants were previously exposed to speaking face stimuli (a face articulating /t/ or /p/ or lexical stimuli embedded into a word, van Linden and Vroomen, 2007), suggesting that abstract representations may mediate such crossmodal effects.

In vision, attractive and contrastive context effects are also found during perception of complex stimuli, including objects (Daelli et al., 2010), bodies (Rhodes et al., 2013), and scenes (Greene and Oliva, 2010). Particularly striking contextual effects occur for faces, affecting the perception of gender, race, expression (Webster et al., 2004), gaze direction (Jenkins et al., 2006), and age (Schweinberger et al., 2010). A striking case in point is facial identity. Here, adaptation to a face typically biases perception of subsequently presented faces away from the adapting stimulus towards a different identity (Leopold et al., 2001). These effects exhibit similar properties as context effects with simple visual stimuli, e.g., a power law dependency on stimulus duration (Leopold et al., 2005). Additionally, both contrastive (Zhao and Chubb, 2001; Anderson and Wilson, 2005; Jiang et al., 2006) and attractive effects (Brooks et al., 2002; Oruc and Barton, 2010) show invariance to various image transformations such as size, position, and view, congruent with the known invariance of higher-level visual representations. Furthermore, facial identity can both be adapted (Hills et al., 2010) and primed (Stevenage et al., 2012) crossmodally using voices, which may involve higher-level multimodal brain areas (Perrodin et al., 2014). Together, these characteristics of attractive and contrastive context effects indicate the involvement of higher-level visual areas, where neurons respond to their preferred category, irrespective of variations in simple stimulus properties.

This then suggests that both in vision and audition, contrastive and attractive effects occur for simple features and for higher-level representations. The temporal properties of context effects for simple stimuli seem to be conserved for complex stimulus processing, indicating that the mechanisms underlying these effects may also be conserved across processing stages. Furthermore, the fact that comparable context effects are observed across and between sensory modalities strongly suggests general computational principles.

### Different Context Effects, Different Mechanisms

**Figure 2:**
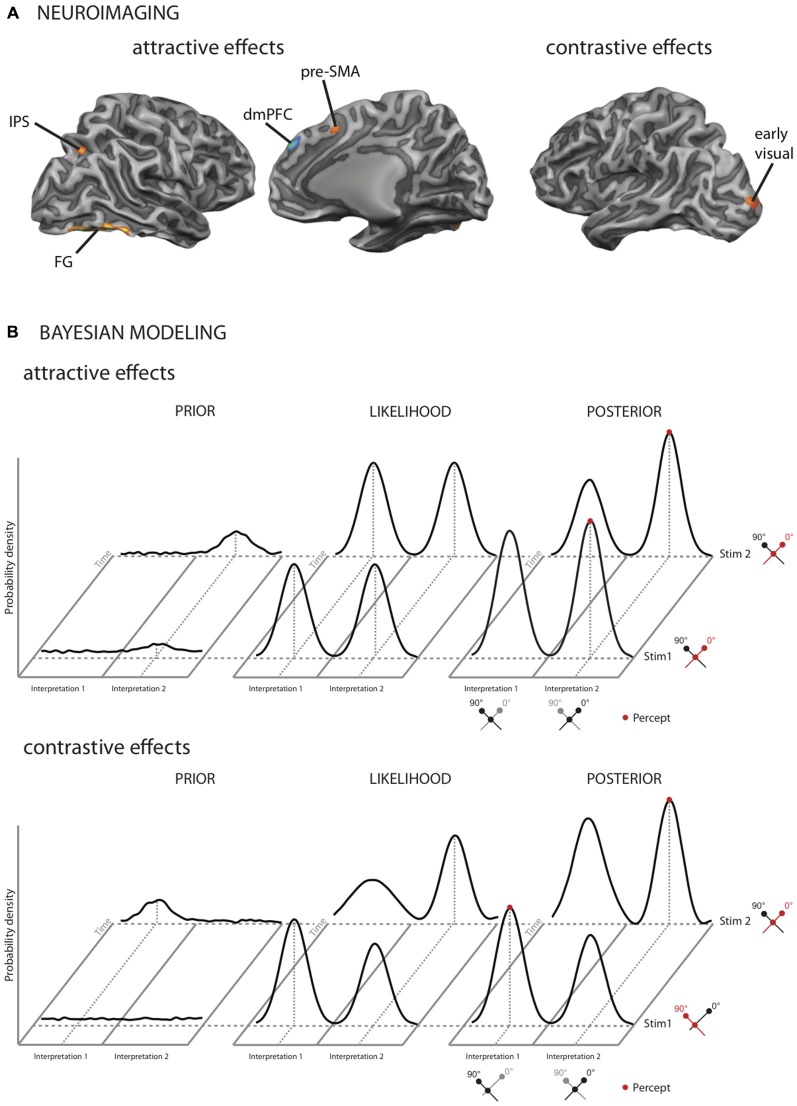
**Neuroimaging results and models for TCEs in vision.** When two multistable stimuli are presented in temporal succession, the initial percept can either systematically repeat, exhibiting an attractive context effect, or switch, exhibiting a contrastive context effect. **(A)** Cortical areas implicated in attractive and contrastive TCEs (Schwiedrzik et al., 2014). In our study, attractive context effects activated a network of fronto-parietal and higher-order visual areas, including the dorsomedial prefrontal cortex (dmPFC), the pre-supplementary motor area (pre-SMA), the intraparietal sulcus (IPS), the anterior insula (not shown), and higher visual areas around the fusiform gyrus (FG). Contrastive TCEs exclusively activated early visual cortex. **(B)** Bayesian model of attractive and contrastive TCEs (Schwiedrzik et al., 2014). Upper panel: attractive context effects result from changes in the prior. In this example, two multistable dot lattices are presented after each other. The perceptual interpretation of these stimuli alternates between two possible orientations (0° or 90°). When the stimuli are completely bistable, as in this example, the likelihood distribution is bimodal with peaks of equal height. When the first dot lattice is presented, a small amount of noise in the prior around one of the possible interpretations may drive up the probability to perceive this interpretation. The maximum in the resulting posterior distribution determines the participant’s percept (red). Subsequently, stimulus 1 provides the temporal context for stimulus 2, increasing the probability to perceive the same orientation again by increasing the prior distribution around the perceived orientation. Lower panel: contrastive effects result from changes in the likelihood function. Following stimulus 1 (i.e., during processing of stimulus 2), which had stronger evidence for interpretation 1, the likelihood function for interpretation 1 is reduced relative to interpretation 2, resulting in a contrastive effect and thus a switch in perception from stimulus 1 to stimulus 2. The reduced likelihood is thought to be a consequence of neuronal adaptation in early sensory areas, which is proportional to the available sensory evidence. **(A,B)** modified from Schwiedrzik et al. (2014), with permission by Oxford University Press.

Given that *identical* stimuli can elicit both types of contextual effects, the question arises whether both effects result from the same or separate neuronal mechanisms. We recently addressed this issue in a functional imaging study (Schwiedrzik et al., 2014) using multistable visual stimuli in which both attractive and contrastive effects were present concurrently but could be separately quantified. The two effects mapped onto distinct cortical networks: higher-order fronto-parietal areas, in particular dorsomedial prefrontal cortex (dmPFC), and higher-order visual areas were active for the attractive effects, while contrastive effects were confined to early visual areas (Figure 2A). Involvement of fronto-parietal areas in attractive effects has also been observed in a recent study on face processing (Kaiser et al., 2013) and for crossmodal context effects (Kilian-Hutten et al., 2011). Furthermore, these neuroimaging results mesh well with behavioral studies: for example, while attractive context effects can transfer beyond the exact location where the context stimulus was presented (Knapen et al., 2009), contrastive effects can be restricted to the retinotopic location of the contextual stimulus (Knapen et al., 2010). This implies that attractive effects arise in brain areas with larger receptive fields. In addition, contrastive effects only occur for test stimuli that are very similar to the context, while attractive effects allow for more variability between context and test (Gepshtein and Kubovy, 2005), in line with the fact that higher brain areas show broader tuning; finally, attractive context effects display a longer time constant than contrastive context effects (Pastukhov and Braun, 2013), mirroring neurons in higher brain areas that integrate over longer time windows (Honey et al., 2012; Chaudhuri et al., 2015).

Together, these results argue for a general dissociation of the underlying mechanisms of attractive and contrastive context effects, and suggest that attractive context effects involve higher-level areas, while contrastive context effects originate in earlier, sensory areas. This processing hierarchy may relate to the function of the proposed computations of attractive and contrastive context effects. In particular, stabilization may be more easily achieved at higher processing stages where neurons exhibit larger receptive fields, longer time constants, and invariance to simple features, while the extraction of new information requires detailed and sensitive representations at earlier sensory processing stages.

## Bayesian Models of Context Effects

Bayesian models provide a general theoretical framework in which attractive and contrastive context effects can be understood at the computational level. Here, perception is framed as an inferential process whereby previous experience is combined with current sensory information. This entails a prior, i.e., a probability distribution of likely percepts given previous experience, and a likelihood function, which can be thought of as the currently available evidence. These two distributions are combined into the posterior distribution through Bayes’ rule, and the maximum of this distribution is what is perceived. The Bayesian framework thus takes temporal context explicitly into account. The different components of Bayesian models allow accommodating the finding that attractive and contrastive effects co-occur and are implemented in different brain regions (Schwiedrzik et al., 2014). We recently proposed one such model (Figure 2B): in this model, attractive contextual effects are conceptualized as resulting from changes in the priors. This is similar to a model by Hohwy et al. (2008), where attractive effects occur as long as the prior distribution is skewed towards the currently dominant percept. We account for contrastive effects by assuming that neural adaptation reduces the available sensory evidence and thus the likelihood function (Stocker and Simoncelli, 2006), linking the Bayesian likelihood function directly to neuronal mechanisms. Alternatively, contrastive effects can be modeled as a temporal drift in the prior distribution, caused by a “hyperprior” that emphasizes change over stability (Hohwy et al., 2008), or by assuming an asymmetric likelihood function that follows the principles of “efficient coding” (Wei and Stocker, 2013). Thus, no matter the specific implementation, the Bayesian framework is compatible with dissociation between attractive and contrastive context effects at the neural level, highlighting its usefulness in understanding the coexistence of these two phenomena.

## Conclusions

In summary, TCEs powerfully modify perception across different senses and stimuli of varying complexity. Neuroimaging data suggest that distinct neural circuits in low-level and high-level brain areas mediate contrastive and attractive effects, respectively. The remarkable similarity of the contextual effects across sensory modalities and stimulus complexity strongly indicates the conservation of mechanisms throughout different sensory systems and levels of processing and raises the possibility that a single computational framework is sufficient to explain both context effects. Bayesian models of perception appear to be useful here as they unify perception, inference, and memory within the same framework. In particular, we show that a Bayesian account can accommodate both attractive and contrastive context effects, at least in vision. However, generalizing this framework to other sensory modalities remains an important challenge for future research. Similarly, more research is needed on the neurophysiological mechanisms, especially of attractive context effects, and directly linking these mechanisms to Bayesian concepts through explicit modeling.

Finding similar context effects across the senses raises the question to what extent modality-specific vs. modality-general brain areas mediate the context effects. At least for simple stimuli, it appears that contrastive effects result from modulations of early modality-specific sensory cortex, while attractive effects recruit fronto-parietal and higher-level modality-specific areas (Kilian-Hutten et al., 2011; Kaiser et al., 2013; Schwiedrzik et al., 2014). The activation of frontal and parietal areas is intriguing, as they are known to respond to multimodal stimuli (Calvert, 2001), making them suitable to convey abstract (modality general) memory representations. Thus, future studies should evaluate whether the same brain areas are active for attractive contextual effects in different senses.

Another interesting question is to what degree attractive and contrastive effects occur between senses. The areal separation described above suggests that contrastive effects might exert weak crossmodal contextual effects, given the recruitment of early unimodal cortex where crossmodal interactions are often weak, while attractive effects that engage higher unimodal areas and associative cortex may lead to greater crossmodal influences. However, crossmodal context effects might be implemented in a different way because the computational demands for unimodal and multimodal representations differ. For instance, multimodal inputs require transformations into a representational format that can make contact with other senses, a demand that is less strong for unimodal representations. Furthermore, it is important to consider that the occurrence of crossmodal influences might be determined less by the neuroanatomical locations of particular processes, but by the extent to which stimuli in different modalities are perceived as arising from the same objects in the physical world. Such a flexible arrangement might enable organisms to behave adaptively in complex multisensory situations typical of everyday life.

## Conflict of Interest Statement

The Reviewer Rachel Denison declares that, despite of being affiliated with the same institution as author Lucia Melloni, the review process was handled objectively. The other authors declare that the research was conducted in the absence of any commercial or financial relationships that could be construed as a potential conflict of interest.
